# Cardiac Structure and Function Is Associated With Hemispatial Neglect Severity

**DOI:** 10.3389/fneur.2021.666257

**Published:** 2021-05-06

**Authors:** Nicole L. Williams, Adrian Suarez, Serban Negoita, Argye E. Hillis, Rebecca F. Gottesman, Michelle C. Johansen

**Affiliations:** Department of Neurology, The Johns Hopkins University School of Medicine, Baltimore, MD, United States

**Keywords:** hemispatial neglect, cardiac, stroke, recovery, left ventricle

## Abstract

**Background:** Hemispatial neglect is a debilitating consequence of right hemispheric ischemic stroke (RIS), with evidence that patient-level factors influence neglect severity. Study objective: Determine if cardiac function is associated with presence and severity of neglect, independent of infarct size.

**Methods:** Two hundred and eighteen non-demented, RIS with cerebral MRI and echocardiography who completed ≥1 of 4 tests evaluating neglect were included. Age- and sex- adjusted Z-scores defined neglect with severity categorized as no neglect, neglect on one or neglect on ≥2 tests. The dependent variable was presence of neglect (multivariable logistic regression), or neglect severity (multinomial logistic regression). The association with left ventricular (LV) structure/function (independent variable) was evaluated using separate nested adjustment models.

**Results:** Patients were on average 61 yo (21–95), female (50%), black (53%), with an ejection fraction of 60% (IQR 20–75%). Fifty eight (27%) had neglect. Each 1 cm increase in LV systolic diameter was associated with a higher relative risk of having neglect on two tests compared to those with no neglect (RRR = 1.83, 95% CI 1.01–3.32), but not after adjusting for education and DWI volume (RRR = 1.68, 95% CI 0.89–3.19). Per 1 cm increase in left atrial (LA) diameter, the relative risk of having neglect on 2 tests vs. no neglect was over two times higher (95% CI 1.04–4.77), but lost significance in the final model (RRR = 1.73, 95% CI 0.76–3.94).

**Conclusions:** We found an association between markers of diastolic dysfunction (enlarging LV, compensatory enlarging LA) and severity of neglect, suggesting that cardiac structure, and function affects not only lesion volume, but also the functional consequences of infarct volume.

## Introduction

Hemispatial neglect is a devastating consequence of right hemispheric ischemic stroke (RIS) and is associated with worse outcomes (1). Several demographic and physiologic factors potentially modulate the degree of impairment from neglect, apart from infarct volume, including age, white matter hyperintensities, extreme hemoglobin levels, dehydration, and cortical hypoperfusion (2–6). Left ventricular (LV) diastolic dysfunction has been associated with death, increased mortality after myocardial infarction, and poor prognosis after ischemic stroke (7). Interestingly, prior work has suggested that the volume of hypoperfusion in RIS is related to the severity of neglect and reduction in the volume of the brain that is hypoperfused, even to a small degree, correlates with degree of early recovery from neglect (8). We have previously demonstrated that subtle changes in LV function are associated with other cerebral pathology, such as infarcts and white matter disease, in adults without clinical stroke (9). It may be that small, incremental changes in the LV might impact the volume of hypoperfused tissue, and thus the manifestation of hemispatial neglect, possibly through a flow phenomenon. We aimed to determine the association of LV structure and function, defined using transthoracic echocardiography (TTE), with presence and severity of hemispatial neglect in a cohort of non-demented RIS patients. We hypothesized that markers of diastolic dysfunction that can be captured on routine, clinical TTE are associated with more severe neglect, and that this association would be independent of infarct volume, an important marker of stroke severity.

## Methods

### Participants

The study was approved by the Johns Hopkins Institutional Review Board, and all patients gave written informed consent. Inclusion criteria were admission to the hospital for acute RIS with diffusion weighted imaging (DWI) and clinically indicated TTE admitted at two academic hospitals (Johns Hopkins Hospital or Johns Hopkins Bayview Hospital). Patients were excluded if they had a cerebral hemorrhage, were non-English speaking, had history of dementia or other neurological disease affecting the brain, uncorrected hearing or visual acuity deficits, ongoing sedation, or were unable to undergo MRI imaging. Patients were approached for written consent while inpatients in the hospital and were evaluated for neglect testing within a time window of 24 h to 5 days from time of RIS. This inclusion criteria reflects that of a parent study, which is an ongoing longitudinal, prospective parent study of the history of neglect in patients with RIS.

### Determination of Neglect

Neglect was determined using four, well-validated tests: horizontal line bisection, line cancellation, Ogden copy scene and gap detection and scored without knowledge of the study hypothesis, infarct volumes, or TTE findings (10). Four different tests to determine neglect were administered as multiple tests are more sensitive to neglect than a single test and might reveal different types of neglect (a focus of the parent study). The horizontal line bisection test entails drawing a horizontal line at the middle of the vertical line. Line cancellation asks patients to cross out all 28 vertical lines on the page. Ogden copy scene involves copying the “Ogden scene,” with the picture including a house, a fence and trees with the total number of components to the picture being 16, yielding a percent error. Finally, in gap detection, the patient is presented with 30 circles and is asked to circle complete circles and cross out circles with the gap, with 10 having a gap on the right, 10 on the left and 10 having no gap. Each test, while possibly revealing different types of neglect, is sufficient to diagnose a global state of neglect (yes/no). Some patients did not complete all of the tests, usually due to time constraints due to other clinical assessments, rehabilitation, visitors, etc.

Z-scores for neglect were defined based on normative data from 58 individuals of similar age who were admitted with TIA or were preoperative coronary artery bypass graft surgical candidates. These patients were chosen as norms due to the fact that they were felt to best represent the vascular risk factor profile that might influence the left ventricle and its function, but did not have an ischemic stroke. Although, using this population for norms might underestimate neglect (if controls showed neglect due to diastolic dysfunction, for example), it would not overestimate neglect or effect the correlations evaluated in this study. Neglect was considered as both a binary and a categorical variable. Neglect was defined as having a Z-score of ≥2 on one of any of the four tests. Neglect severity was then categorized for those who completed at least two tests as: no neglect, neglect on 1 or neglect on ≥2 or more tests, as previously reported (11). The data are available from the first author upon reasonable request.

### Echocardiography Assessment

TTE was obtained during admission at the discretion of the inpatient treatment team, but is fairly routine at the recruiting institution. All TTEs were performed by accredited technicians, with reports generated by board-certified cardiologists, and using the Philips IE33 ultrasound in standard 2D-analysis. The following variables were recorded from each patient's TTE report according to the pre-specified analysis: LV ejection fraction (LVEF, %), LV ventricular diastolic diameter (cm), LV systolic diameter (cm), Left atrial (LA) diameter (cm) and mitral E to A ratio. Mitral E and A point velocities are components of the mitral inflow signal to assess diastolic filling.

### Ischemic Stroke Volume

All patients underwent a brain 3-T MRI with contrast. Infarct volume was defined using DWI with manual lesion tracing performed using either ImageJ^©^ software or MRICron^©^. Such techniques have been well-validated for calculating infarct volumes (12). Technicians measuring infarct volume were masked to patient characteristics, neglect scores and TTE variables.

### Statistical Methods

Primary dependent variables were presence of neglect (vs. none) or severity of neglect as defined above. Pre-specified TTE variables (primary independent variables) were each evaluated descriptively and in univariate analyses in association with the outcome of interest using crude logistic regression models. Subsequent models then explored the association between presence of neglect (multivariable logistic regression), and then severity of neglect (multinomial logistic regression) with each specified TTE variable in separate analyses, with the same nested adjustment models. Each of the LV variables were considered as separate independent variables, in separate models and were pre-specified prior to the analysis. Covariates were obtained at the time of consent and were chosen based on the possibility of confounding the association between neglect and the TTE variables. In a sensitivity analysis, the Bonferroni correction was applied to adjust for the potential for multiple comparisons.

The nested adjustment models were as follows: Model 1 adjusted for sex, race, and age; Model 2 for Model 1 plus atrial fibrillation, and Model 3 for Model 2 plus education and DWI volume. Age was centered at 65 years old and considered in increments of 10 years. DWI volume was centered at the mean and considered in increments of 10 cm^3^. Education was dichotomized into 12th grade and over vs. below 12th grade. LVEF is reported per 10% increase.

All statistical analyses were performed using Stata v14.1. Two-sided *p* < 0.05 was considered statistically significant.

## Results

### Demographics

Two hundred and eighteen patients met inclusion criteria (Table 1) and 212 patients completed at least 2/4 of the assessments for neglect. One hundred and sixty patients did not have any evidence of neglect on any assessment, while 58 participants had evidence of neglect on at least one test. Among those who had neglect, 27 (47%) completed all four tests, while 81 (51%) without neglect completed all four tests. There was a significant difference in age (≥65 vs. <65 yo) among those who had neglect, vs. those who did not have neglect (χ = 6.25, *P*-value 0.01).

**Table 1:** Characteristics of the cohort (*N* = 218)^*^.

Age [mean years (SD)]	61.2 (15)
Female sex	109 (50)
Black race	115 (53)
Below a 12th grade education	70 (32)
Atrial fibrillation	28 (13)
Hypertension	166 (76)
Hyperlipidemia	85 (39)
Current smokers	71 (33)
Prior stroke	69 (32)
History of coronary artery disease	37 (17)
Heart failure with reduced ejection fraction (EF ≤ 40%)	17 (8)

* *All characteristics (except age) labeled as N (%). Education dichotomized into ≥12th vs. <12th grade. All demographics defined at time of admission*.

### Association Between Neglect and Cardiac Structure and Function

There was no significant association between any of the TTE variables of interest and presence of neglect (Table 2). When considering the relative risk of more severe neglect, a 1 cm increase in LV systolic diameter was associated with a higher relative risk of having neglect on two tests compared to those with no neglect for a participant 65 yo and similar risk factors (RRR = 1.83, 95% CI 1.01–3.32, Model 2, Table 3), but not after accounting for education and DWI volume (RRR = 1.68, 95% CI 0.89–3.19, Model 2). A 1 cm larger LA diameter was associated with approximately twice the risk of having neglect on two tests vs. no neglect (RRR = 2.23, 95% CI 1.04–4.77, Model 2), but again lost significance in the final model (RRR = 1.73, 95% CI 0.76–3.94). When considering the results after adjustment for the potential for multiple comparisons, the effect estimates were no longer statistically significant (*p*-value ≤ 0.01).

**Table 2:** Multivariable logistic regression demonstrating the odds ratio of neglect per unit increase in the transthoracic echocardiogram variable (*N* = 218).

**Cardiac parameter**	**Model 1 OR (95% CI)**	**Model 2 OR (95% CI)**	**Model 3 OR (95% CI)**
Left ventricular ejection fraction (LVEF %)^*^	1.01 (0.98, 1.04)	1.01 (0.98, 1.04)	1.01 (0.98, 1.04)
Left ventricular diastolic diameter (cm)	1.01 (0.63, 1.60)	1.00 (0.63, 1.60)	0.94 (0.59, 1.52)
Left ventricular systolic diameter (cm)	1.13 (0.76, 1.66)	1.12 (0.76, 1.66)	1.07 (0.72, 1.60)
Left atrial diameter (cm)	1.28 (0.82, 2.00)	1.24 (0.79, 1.95)	1.13 (0.71, 1.82)
E/A ratio	0.95 (0.58, 1.55)	0.91 (0.53, 1.58)	0.88 (0.51, 1.53)

* *LVEF centered at 55 with 10-percent interval increase*.

*Adjustment models: Model 1: Age (centered at 65), sex, race. Model 2: Model 1+ atrial fibrillation. Model 3: Model 2+ education (≥12th vs. <12th grade), diffusion weighted image stroke volume (centered at 15 cm^3^)*.

**Table 3:** Multinomial logistic regression demonstrating the relative risk ratios of neglect, vs. no neglect, per unit increase in the transthoracic echocardiogram variable (*N* = 212).

**Cardiac parameter**	**Neglect on 1 test**			**Neglect on** **≥2 tests**		
	**Model 1 RRR (95% CI)**	**Model 2 RRR (95% CI)**	**Model 3 RRR (95% CI)**	**Model 1 RRR (95% CI)**	**Model 2 RRR (95% CI)**	**Model 3 RRR (95% CI)**
Left ventricular ejection fraction (LVEF %)^*^	1.00 (0.97, 1.04)	1.00 (0.97, 1.04)	1.00 (0.97, 1.04)	0.99 (0.95, 1.04)	0.99 (0.95, 1.04)	0.99 (0.95, 1.04)
Left ventricular diastolic diameter (cm)	0.71 (0.40, 1.29)	0.69 (0.38, 1.26)	0.67 (0.37, 1,25)	1.91 (0.95, 3.85)	1.91 (0.96, 3.84)	1.75 (0.84, 3.67)
Left ventricular systolic diameter (cm)	0.89 (0.54, 1.46)	0.87 (0.53, 1.44)	0.86 (0.52, 1.43)	1.84 (1.01, 3.37)	1.83 (1.01, 3.32)	1.68 (0.89, 3.19)
Left atrial diameter (cm)	0.80 (0.45, 1.42)	0.75 (0.42, 1.35)	0.73 (0.41, 1.33)	2.09 (1.01, 4.35)	2.23 (1.04, 4.77)	1.73 (0.76, 3.94)
E/A ratio	0.99 (0.58, 1.69)	0.96 (0.53, 1.72)	0.95 (0.53, 1.72)	0.86 (0.36, 2.08)	0.80 (0.29, 2.18)	0.80 (0.29, 2.18)

* *LVEF centered at 55 with 10-percent interval increase*.

*Adjustment models: Model 1: Age (centered at 65), sex, race. Model 2: Model 1+ atrial fibrillation. Model 3: Model 2+ education (≥12th vs. <12th grade), diffusion weighted image stroke volume (centered at 15 cm^3^)*.

## Discussion

We found an association between markers of diastolic dysfunction (enlarging LV, compensatory enlarging LA) and severity of neglect, defined as more frequent neglect on four standard tests. Our findings are clinically relevant as hemispatial neglect can complicate acute rehabilitation therapy following a stroke, and is frequently associated with other cognitive deficits (13), so identifying contributing factors would influence patient care. Diastolic dysfunction is established as a major cause of heart failure in patients with a normal LV ejection fraction, and stands as an independent predictor of all-cause mortality (14). It may be that normalizing or optimizing diastolic function, particularly in patients with even more gross findings of dysfunction, rather than just subtle changes, could lead to improved functional outcomes.

Apart from the presence of atrial fibrillation, or acute LV thrombus, cardiac findings on TTE do not currently change stroke care. It is important to consider that there may be meaningful information that might be garnered from the TTE that is not currently used in the care of the stroke patient, particularly considering that this test is usually routine for comprehensive stroke centers in order to evaluate the cardiovascular system as recommended by the American Stroke Association guidelines. There were many LV variables that could have been chosen for analysis, including the E/E' ratio and the presence of tricuspid regurgitation. We chose to include those variables that are most generalizable based on those that are most frequently collected on routine stroke echo, and those that have the most evidence behind the association between their function and stroke outcome. We anticipate a time when the cardiac evaluation will be more tailored to the ischemic stroke patient; for example, further evaluating the function of the left atrium in a patient with cardioembolic stroke or embolic stroke of unknown source, while focusing on the LV and diastolic function for a patient with hemispatial neglect, may be informative.

Here we show that incremental changes in LV function impact not only infarct volume, but also its functional consequences, measured by severity of neglect. If hemispatial neglect does not recover after a stroke, it is associated with poorer outcomes (15). As a result, even minor improvements in function would be meaningful. The fact that the observed association lost significance after adjusting for infarct volume may reflect lack of power, but might reflect partial mediation by infarct volume, and thus stroke severity. Work in this cohort is ongoing and represents an opportunity to consider these associations in the future with more participants, thereby increasing power.

We readily acknowledge limitations to our study. This was a cross-sectional analysis and we cannot comment on causality or mechanism. We required that participants complete at least one test to be eligible, and it may be that those with more severe neglect were unable to complete any tests, or fewer tests, potentially meaning our population had milder neglect. We would anticipate, however, that including those with more severe neglect would strengthen associations, rather than bias toward the null. Furthermore, the number of tests completed was more often constrained by time limitations due to other clinical assessments, rehabilitation, and so on. Even patients with severe neglect were generally able to complete line bisection and line cancellation. When performing a correction for the potential for multiple comparisons, our effect estimates were no longer statistically significant. We chose our independent variables a-priori and ran all estimates in separate models, but it may be that our findings are the result of chance.

In conclusion, we have shown the possible importance of diastolic function in outcomes of RIS, specifically hemispatial neglect. Further, study of the possible mechanisms behind this association are needed.

## Data Availability Statement

The raw data supporting the conclusions of this article will be made available by the authors, without undue reservation.

## Ethics Statement

The study involving human participants were reviewed and approved by the Johns Hopkins Institutional Review Board in accordance with the local legislation and institutional requirements. The patients/participants provided their written informed consent to participate in this study.

## Author Contributions

All authors listed have made a substantial, direct and intellectual contribution to the work, and approved it for publication.

## Conflict of Interest

The authors declare that the research was conducted in the absence of any commercial or financial relationships that could be construed as a potential conflict of interest.
